# Classes of Oppositional Defiant Disorder Behavior in Clinic-referred Children and Adolescents: Concurrent Features and Outcomes: Classification Des Comportements Dans le Trouble Oppositionnel Avec Provocation Chez Des Enfants et des Adolescents Aiguillés à Une Clinique: Caractéristiques Co-occurrentes et Résultats

**DOI:** 10.1177/0706743720974840

**Published:** 2020-11-26

**Authors:** Peter J. Roetman, Berend M. Siebelink, Robert R. J. M. Vermeiren, Olivier F. Colins

**Affiliations:** 1 The Department of Child and Adolescent Psychiatry, 4501 Leiden University Medical Center, Oegstgeest, the Netherlands; 2 Youz, Parnassia Group, The Hague, the Netherlands; 3 The Department of Special Needs Education, Ghent University, Belgium; 4 The Center for Criminological and Psychosocial Research, Örebro University, Sweden

**Keywords:** oppositional defiant disorder, irritable mood, clinical decision-making, diagnosis-related groups

## Abstract

**Objective:**

Oppositional defiant disorder (ODD) consists of irritable and oppositional behaviors, both of which are associated with different problems. However, it is unclear whether irritability and oppositionality enable classification of clinic-referred children and adolescents into mutually exclusive groups (e.g., high in oppositionality, low in irritability), and whether this classification is clinically meaningful.

**Method:**

As part of a clinical protocol, ODD behaviors were assessed at referral through a comprehensive diagnostic interview and questionnaire. Parent- and teacher-reported ODD of 2,185 clinic-referred 5- to 18-year-olds (36.9% females) were used in latent class analysis. Resulting ODD classes were compared, concurrently at referral, and, longitudinally at the end of the diagnostic and treatment process, on various clinically relevant measures that were completed by various informants, including mental health problems, global functioning, and *Diagnostic and Statistical Manual of Mental Disorders* (*DSM*) classifications.

**Results:**

Three classes emerged with high, moderate, and low levels of both irritability and oppositionality. At referral, the high class experienced the highest levels of mental health problems and *DSM* classifications. Importantly, all ODD classes defined at intake were predictive of diagnostic and treatment outcomes months later. Notably, the high class had higher rates of clinician-based classifications of ODD and conduct disorder, and the lowest levels of pre- and posttreatment global functioning. Additionally, the low class exhibited higher rates of generalized anxiety disorder and fear disorders.

**Conclusions:**

Irritability and oppositionality co-occur in clinic-referred youths to such an extent that classification based on these behaviors does not add to clinical inference. Instead, findings suggest that the overall ODD severity at referral should be used as a guidance for treatment.

## Abstract (fr)

**Objectif:**

Le trouble oppositionnel avec provocation (TOP) consiste en des comportements irritables et oppositionnels, qui sont tous deux associés à des problèmes différents. Cependant, il n’est pas certain que l’irritabilité et l’opposition permettent la classification d’enfants et d’adolescents aiguillés à une clinique dans des groupes mutuellement exclusifs (p. ex., forts en opposition, faibles en irritabilité), et que cette classification soit cliniquement significative.

**Méthode:**

Dans le cadre d’un protocole clinique, les comportements du TOP ont été évalués lors de l’aiguillage à l’aide d’une entrevue diagnostique détaillée et d’un questionnaire. Les TOP rapportés par les parents et les enseignants de 2185 enfants et adolescents de 5 à 18 ans aiguillés à la clinique (36,9% de sexe féminin) ont été utilisés dans l’analyse de classes latentes. Les classes de TOP résultantes ont été comparées, de façon concurrentielle lors de l’aiguillage, puis longitudinalement au terme du processus diagnostique et de traitement, par diverses mesures cliniquement pertinentes qui ont été appliquées par différents informateurs, notamment les problèmes de santé mentale, le fonctionnement général, et les classifications du DSM.

**Résultats:**

Trois classes se sont dégagées qui portaient des niveaux élevés, modérés et faibles tant d’irritabilité que d’opposition. À l’aiguillage, la classe de niveau élevé présentait les niveaux les plus élevés de problèmes de santé mentale et de classifications du DSM. À noter, toutes les classes de TOP définies au départ étaient prédictives du diagnostic et des résultats du traitement des mois plus tard. Notablement, la classe de niveau élevé avait des taux plus élevés de classifications de TOP et de trouble des conduites selon un clinicien, et les niveaux les plus faibles de fonctionnement général prétraitement et posttraitement. En outre, la classe de niveau faible présentait des taux plus élevés de trouble d’anxiété généralisée et de troubles phobiques.

**Conclusions:**

L’irritabilité et l’opposition co-occurrent chez des adolescents aiguillés à une clinique à tel point que la classification basée sur ces comportements n’ajoute pas à l’inférence clinique. Les résultats suggèrent plutôt que la gravité générale du TOP lors de l’aiguillage devrait servir de guide pour le traitement.

## Introduction

*Diagnostic and Statistical Manual of Mental Disorders* (*DSM*)-defined oppositional defiant disorder (ODD) is characterized by a pattern of problem behaviors ranging from anger and temper tantrums to arguing and vindictiveness.^1^ In addition to this heterogeneity in ODD symptomatology, children with ODD differ greatly in co-occurring mental health problems and prognosis.^2–4^ In order to gain further insight into this heterogeneity, efforts to distinguish between types of ODD behavior have shown that a differentiation can be made between at least two dimensions: an irritable dimension, consisting of touchy and angry behavior, and an oppositional dimension, consisting of hurtful and headstrong behavior.^5,6^ Irritability is mainly associated with affective problems, especially depression and anxiety,^7,8^ whereas oppositionality is correlated with symptoms of attention deficit hyperactivity disorder (ADHD) and conduct disorder (CD), as well as violent and nonviolent delinquency.^7^ Some evidence suggests that the oppositional dimension can be divided further into a hurtful dimension, consisting of vindictive and spiteful behaviors, and a headstrong dimension, characterized by arguing, defiance, blaming, and annoying behavior.^9^ Yet, it is still unclear which dimensional approach (i.e., differentiating between two or three dimensions) is most useful for applied clinical purposes.

Crucially, it remains unclear to what extent distinct ODD dimensions enable classification of clinic-referred children and adolescents into mutually exclusive groups (e.g., children who are only high in one ODD dimension vs. children who are high in two or three ODD dimensions). The majority of prior studies explored this issue in community samples,^10–14^ with three notable exceptions. One study used latent class analysis (LCA) to assign 177 7- to 12-year-old clinic-referred boys to separate classes on the basis of parent-reported ODD symptoms.^15^ Based on this data-driven analysis, three classes emerged; one class comprised of boys low in oppositionality and irritability (low ODD class); a second class high in oppositionality, but low in irritability (oppositional ODD class); and a third class high in both oppositionality and irritability (combined ODD class). The prognostic usefulness of the classes was also supported; the combined ODD class had the highest levels of future self-reported anxiety and depression in adolescence and was highest in adult neuroticism and depression. Unfortunately, differences between the oppositional ODD and the low ODD class were not reported.^15^ A second study performed LCA in a sample of 158 detained male juvenile offenders,^16^ a population hallmarked by severe psychopathology.^17,18^ Besides the aforementioned classes, a fourth class was revealed, characterized by substantial irritability, but low oppositionality (irritable ODD class). Cross-sectionally, the irritable and combined ODD classes were related to suicidality and comorbid affective/anxiety disorders. The irritable ODD class was at risk of criminal reoffending, even when controlling for CD.^16^ The third study used theory-driven classifications to assign 1,160 6- to 18-year-old clinic-referred youths to angry/irritable symptoms (AIS), primarily noncompliant symptoms (NS), and control groups.^19^ The AIS group showed the highest levels of concurrent parent- and teacher-reported anxiety, mood, and conduct symptoms, while the NS and control groups showed moderate and low levels of symptoms, respectively. In sum, prior work consistently shows that children and adolescents in the combined ODD class experience substantial concurrent problems, while the differentiating capabilities of the oppositional and irritable classes are less clear. Furthermore, several important aspects that determine the clinical usefulness of these classes, like outcomes of the diagnostic process (e.g., clinician-based *DSM* classifications) or treatment, have not been studied.

This is the first study to investigate the viability of ODD classes for actual clinical inference; using data that were collected as part of a clinical protocol, starting at time of referral, and spanning the diagnostic process and treatment. Also, whereas prior work with community and clinic-referred samples merely considered the presence of ODD symptoms, this study will be the first to account for *DSM*-defined criteria of duration (≥ 6 months) and impairment in developmental contexts (e.g., family, friends). To facilitate comparison with most prior work,^10–13,15,16^ LCA was used to assign children and adolescents to ODD classes. This data-driven analytical approach enabled us to investigate differences in ODD symptom profiles without committing ourselves to a priori choices about the number (2 or 3) and the content (e.g., noncompliance only) of ODD dimensions. Contrary to prior work that relied on relatively small samples^15,16^ the current study used a large sample of clinic-referred children and adolescents (*N* = 2,185), guaranteeing optimal model estimation.^20^ We broadly expect to identify low, oppositional, and combined ODD classes, with youths in the latter class exhibiting the lowest level of concurrent and future functioning. Yet, we do not rule out the existence of an irritable ODD class.^16^ An oppositional class would show substantial rates of conduct problems as well as ADHD but relatively low levels of affective problems. Conversely, an irritable class would show considerable levels of affective problems but low conduct problems and rates of ADHD.

## Method

### Participants and Procedure

This study used data that were collected as an integral part of a clinical protocol at a center for child and adolescent psychiatry between October 2008 and October 2017. The center is located in a predominantly urban area with moderate to high socioeconomic status in the western Netherlands. The sample consisted of 5- to 18-year-old youths of predominantly Dutch European descent who were referred for various psychiatric problems, spanning from anxiety and depression to neurodevelopmental disorders. Youths with suspected low intelligence were referred to other institutions. Parents and youths were informed that their anonymized data could be used for scientific purposes at time of admission. To be eligible for admission and subsequent aftercare, parents and, if applicable, teachers were required to complete the Development and Well-Being Assessment at referral (DAWBA; see Measures).^21^ The care provided was diverse, ranging from diagnostics, to various inpatient and outpatient treatment programs.

For 3,362 youths, DAWBA reports were available from parents or teachers. Because diagnostic assessment of youths emphasizes information from multiple informants,^22,23^ only youths for whom DAWBA ODD parent- or teacher information was available were selected (excluding 387 youths). Next, we excluded 790 participants for whom parents did not report on all ODD symptoms (because they did not reach the DAWBA ODD screening threshold; see Measures). Thus, in total, 2,185 youths (36.9% female) between the ages of 5 and 18 years (*M* = 9.96, *SD* = 3.22) were included. Due to missing values, the number of participants used for group comparisons will be slightly lower (≤2,041) than those in the model-based clustering analyses (*N* = 2,185).

### Measures

#### Clustering variables

*DSM*-IV defined ODD behaviors or symptoms were measured by the Dutch parent and teacher versions of the DAWBA, a widely used computerized diagnostic interview.^21^ The Dutch DAWBA version separates the *DSM* symptom “vindictive and spiteful” into two different questions (see Table S1), resulting in a total of 9 ODD symptoms. According to the *DSM*, we focused on clinically significant levels of the 9 ODD symptoms, meaning we considered symptoms which are oft-occurring (“occurs a lot more than in other children”), persistent (“present for 6 months or longer”), and cause functional impairment in 1 or more developmental contexts. Finally, the 9 DAWBA ODD symptoms will be used as clustering variables in LCA to assign youths to mutually exclusive classes. Consistent with recommendations to use multiple informants,^1^ the highest score from the parent and teacher for each ODD symptom was used.^24^ This means that if at least 1 informant indicated an ODD symptom to be present, persistent, and impairing, the ODD symptom was indicated as present. Details about the use of the DAWBA ODD symptoms are found in Supplement 1.

#### Variables for cluster comparisons at referral

Parent, teachers, and if applicable, youths completed the Strengths and Difficulties Questionnaire (SDQ) as an index of dimensionally assessed mental health problems (emotional problems and hyperactivity) and other problems (peer problems and prosocial behavior).^25^ Additionally, and in line with recommendations^26^ and prior work,^23^ we used the *DAWBA computer-generated DSM disorder categories* “depressive disorders” (referring to the presence of major depressive disorder, dysthymic disorder, and/or depressive disorder not otherwise specified) and “fear disorders” (referring to the presence of separation anxiety disorder, panic disorder agoraphobia specific, and/or social phobia).

#### Variables for longitudinal cluster comparisons

As an index of categorically assessed mental health problems, we relied on diagnoses of *DSM*-IV-defined psychiatric disorders that were determined by a multidisciplinary team at the end of a diagnostic process, conform clinical diagnostic guidelines. A main advantage of clinical classifications by a multidisciplinary team over parent- and teacher-reported classifications is the ability of clinicians to weigh several constellations of symptoms against one another to establish which symptoms (i.e., clinical classification[s]) are likely to be the main problem. Another important advantage is their ability to pick up symptoms that are difficult to detect (e.g., autistic symptoms) by nontrained raters (e.g., parents and teachers). These multidisciplinary evaluations took place on average 3.81 months (*SD* = 3.34) after referral. Any clinical classification, not just primary classifications, were included in the analyses. We also collected *DSM*-based Global Assessment Functioning (GAF) scores at the beginning and end of treatment as an index of clinician-rated global functioning (see Supplement 1 for details).

### Data Analyses

Table 1 provides descriptive information for all variables. According to most prior work on ODD subtypes,^10,11,12,13,15,27^ LCA was performed using the 9 ODD symptoms as clustering variables. LCA is a data-driven model-based clustering technique enabling differentiation between classes of youths with various constellations of ODD symptoms. Specifically, LCAs provide a probability of endorsement of an ODD symptom within a class, with a value of 1 indicating a 100% probability of item endorsement (e.g., youths in this class are always reported to have temper tantrums), while a 0 indicates a 0% chance of endorsement. LCA also provides per individual the most probable class to which he or she belongs. In the LCA, it was assessed whether gender and/or age should be included as covariates. These covariates were deemed important because of ODD’s gender ^28^ and developmental differences (e.g., ODD rarely develops after early adolescence).^29^ To test whether ODD classes differed in dimensionally and categorically assessed variables, analyses of variance (ANOVAs) and logistic regressions were performed. Finally, to examine whether ODD classes differed in pre- and posttreatment functioning repeated measures ANOVAs were performed, with pre- and posttreatment GAF scores as within-subjects factor and ODD class as between-subjects factor. To account for multiple testing, we used *P* < 0.01 as an indicator of statistical significance. Cohen’s *d*s were calculated for continuous measures. Two-tailed tests were used in all analyses. LCAs were conducted in Mplus version 8,^30^ all other analyses in SPSS version 25.^31^

**Table 1.** Descriptive Statistics for Youths with Parent- and Teacher-reported Oppositional Defiant Disorder Data.

	Variable	Mean (*SD*)	Range
Latent class analysis data (*N* = 2,185)	Youth’s gender male (PR), *n* (%)	1,378 (63.1%)	0 to 1
	Age in years (PR)	9.96 (3.22)	5 to 18
	ODD criteria (PR, TR)	3.29 (3.30)	0 to 9
	Irritable ODD criteria (PR, TR)	1.25 (1.27)	0 to 3
	Oppositional ODD criteria (PR, TR)	2.03 (2.20)	0 to 6
Cross-sectional data (*n* = 2,164)	Strengths and Difficulties Questionnaire Scales (PR, TR, SR)		
	Total problems	20.30 (5.30)	3 to 38
	Emotional problems	5.81 (2.54)	0 to 10
	Conduct problems	4.22 (2.00)	0 to 10
	Hyperactivity	7.12 (2.40)	0 to 10
	Peer problems	3.97 (2.25)	0 to 10
	Prosocial behavior	7.05 (1.99)	0 to 10
	DAWBA computer-generated DSM classifications (PR, TR, SR)		
	Oppositional Defiant Disorder, *n* (%)	959 (44.3%)	0 to 1
	Conduct disorder, *n* (%)	219 (10.1%)	0 to 1
	ADHD, *n* (%)	848 (39.2%)	0 to 1
	Depressive disorders, *n* (%)	333 (15.4%)	0 to 1
	Generalized anxiety disorder, *n* (%)	355 (16.4%)	0 to 1
	Fear disorders, *n* (%)	451 (20.8%)	0 to 1
	Autism spectrum disorder, *n* (%)	99 (4.6%)	0 to 1
Longitudinal data (*n* = 2,041)	Multidisciplinary team-based *DSM* classifications (CR)		
	Oppositional defiant disorder, *n* (%)	177 (8.7%)	0 to 1
	Conduct disorder, *n* (%)	69 (3.4%)	0 to 1
	ADHD, *n* (%)	755 (37.0%)	0 to 1
	Depressive disorders, *n* (%)	137 (6.7%)	0 to 1
	Generalized anxiety disorder, *n* (%)	92 (4.5%)	0 to 1
	Fear disorders, *n* (%)	61 (3.0%)	0 to 1
	Autism spectrum disorder, *n* (%)	486 (23.8%)	0 to 1
	Global Functioning (CR)		
	Global Assessment Functioning pretreatment^a^	52.49 (6.66)	6 to 80
	Global Assessment Functioning posttreatment^b^	54.58 (7.32)	5 to 80

*Note*. ADHD = attention deficit hyperactivity disorder; CR = clinician-rated; DAWBA = Development and Well-being Assessment; *DSM* = *Diagnostic and Statistical Manual of Mental Disorders*; ODD = oppositional defiant disorder; PR = parent-reported; SR = self-reported; TR = teacher-reported.

^a^ *n* = 1,997.

^b^ *n* = 1,630, pairwise *n* = 1,628.

## Results

### Identification of Classes

**Figure 1.**
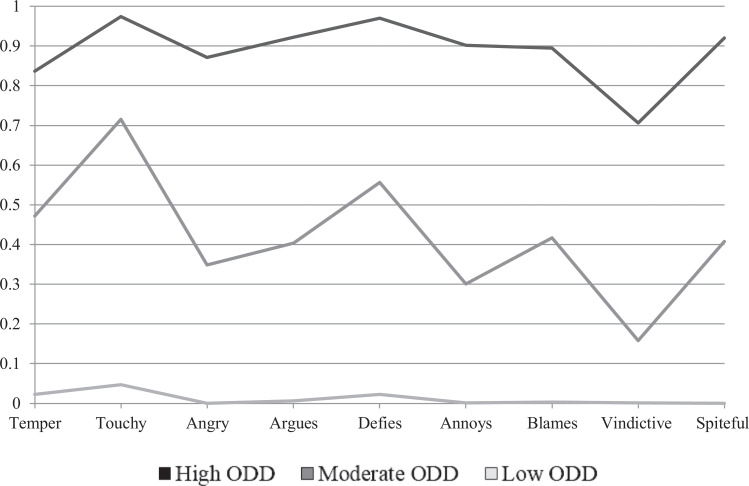
Three-class *Diagnostic and Statistical Manual of Mental Disorders* solution for parent- and teacher-reported oppositional defiant behavior of the Development and Well-Being Assessment (DAWBA). *Note*: *N* = 2,185. High ODD = 576 (26.4%); moderate ODD = 698 (31.9%); low ODD = 911 (41.7%). ODD = oppositional defiant disorder.

Table S4 shows that the LCA indicated a 3-class solution to be the best fit (see Supplement 2 for details)^i^. Additional analyses revealed it was unnecessary to control for age and gender (Supplement 2 and Table S5). Figure 1 shows that participants were assigned to 1 class high in both oppositionality and irritability with a high probability of ODD (high ODD class; 25.8% of total sample), 1 class low in both behaviors and a low probability of ODD (low ODD class; 34.7%), and 1 class with moderate levels of oppositionality and irritability and a moderate probability of ODD (moderate ODD class; 39.4%).

### Class Comparisons: Concurrent Features at Referral

#### Dimensionally assessed mental health and other problems

**Figure 2.**
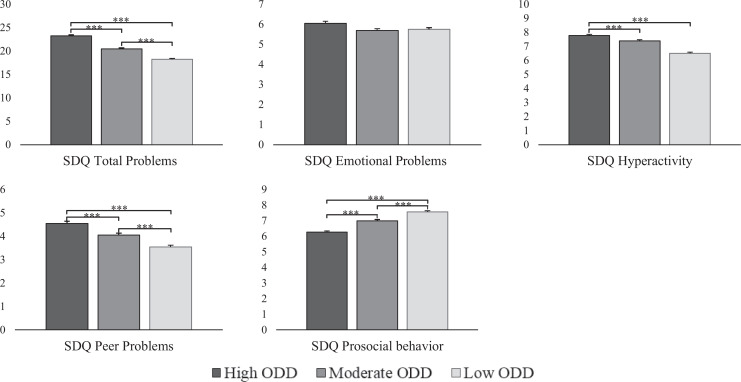
Differences of the oppositional defiant disorder classes on highest prevailing parent- self- and teacher-reported strength and difficulties questionnaire scores. *Note: N* = 2164. ***P < 0.001. **P < 0.01.

Figure 2 shows that participants in the high ODD class had significantly higher levels of total, hyperactivity, and peer problems, and lower levels of prosocial behavior than the two other classes (range *d*: 0.17 to 1.00) with the exception of emotional problems. Furthermore, the moderate class functioned worse than the low ODD class in terms of total problems, hyperactivity, peer problems, and prosocial behavior (range *d*: 0.23 to 0.47) but had comparable levels of emotional problems (see Table S6 for descriptives).

#### Categorically assessed mental health problems

**Figure 3.**
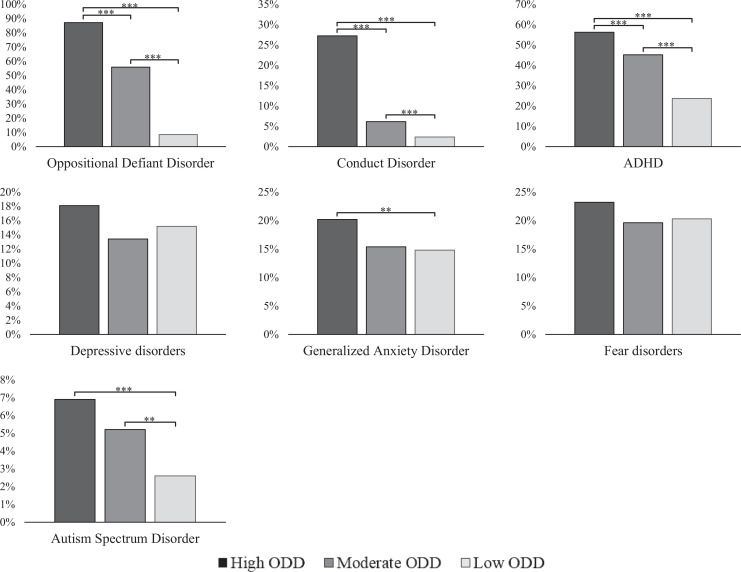
Prevalence of DAWBA classifications and differences between parent- self- and teacher-reported oppositional defiant disorder classes. *Note: N* = 2,164. ADHD = attention deficit hyperactivity disorder. The *y*-axis indicates the disorder rate in each respective class. ***P < 0.001. **p < 0.01.

Figure 3 shows that the rates of DAWBA computer-generated classifications of ODD, CD, and ADHD were higher in the high ODD class as compared to the other two ODD classes (see Table S7 for descriptives). The high ODD class also was higher in DAWBA computer-generated classifications of autism spectrum disorders (ASD) and GAD than the low ODD class, while both classes did not differ in depressive and fear disorders. The moderate ODD class was higher than the low ODD class in ODD, CD, ADHD, and ASD but were equal in terms of internalizing disorders (i.e., GAD, depression, and fear disorders).

### Class Comparisons: Longitudinal Features

#### Categorically assessed mental health problems

**Figure 4.**
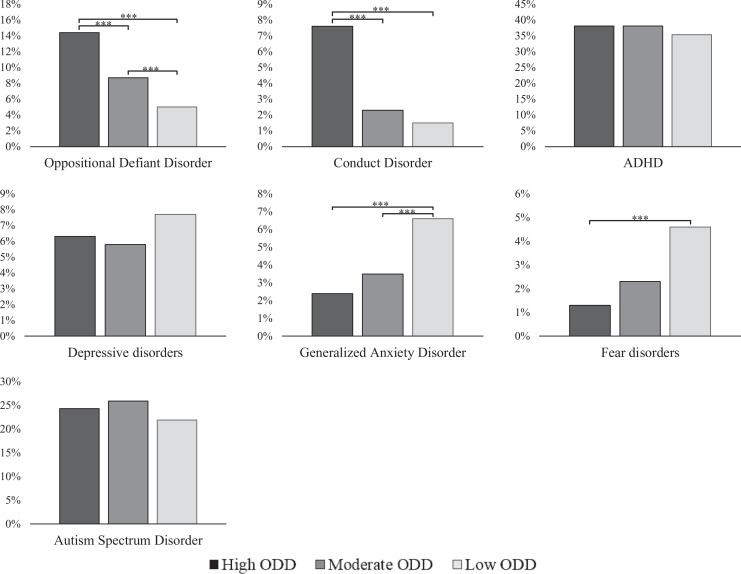
Prevalence of clinical classifications and differences between the oppositional defiant disorder classes. *Note. N* = 2,041. ADHD = attention deficit hyperactivity disorder. The *y*-axis indicates the disorder rate in each respective class. ***P < 0.001.**P < 0.01.

In terms of multidisciplinary team-based classifications, the high ODD class had significantly higher rates of ODD and CD than the 2 other ODD classes (Figure 4; see Table S8 for descriptives). Further, compared to the Low ODD class, both the high and moderate ODD classes had significantly lower rates of GAD, the high ODD class had a lower rate of fear disorders, whereas the moderate ODD class had a higher rate of ODD than the low ODD class. No class differences emerged in rates of ADHD, depressive disorders, and ASD.

#### Pre- and posttreatment functioning

The three ODD classes differed in terms of clinician-rated GAF scores at both the beginning, *F*(2, 1994) = 19.58, *P* ≤ 0.001, range *d*: 0.35 to 0.15, and end of treatment, *F*(2, 1627) = 22.22, *P* ≤ 0.001, range *d*: 0.43 to 0.18, with the high ODD class showing the highest impairment (start of treatment: *M* = 51.14, *SD* = 6.02; end of treatment: *M* = 52.85, *SD* = 6.42), followed by the moderate (start of treatment: *M* = 52.39, *SD* = 6.30; end of treatment: *M* = 54.44, *SD* = 7.80), and low classes (start of treatment: *M* = 53.43, *SD* = 7.14; end of treatment: *M* = 55.81, *SD* = 7.25). All classes increased in functioning during treatment, *F*(1, 1625) = 207.56, *P* ≤ 0.001, ηp^2^ = 0.11, though these changes were independent of class membership, *F*(2, 1625) = 1.20, *P* = 0.30.

## Discussion

Model-based clustering analyses in clinic-referred youths showed three distinct ODD classes: high ODD (high in irritability and oppositionality), moderate ODD (moderate levels of irritability and oppositionality), and low ODD (low in irritability and oppositionality). We could not find children and adolescents who were solely high in oppositionality (oppositional ODD class) or solely high in irritability (irritable ODD class). Instead, the overall severity of the ODD symptoms differentiates between individuals, suggesting that classification of clinic-referred youths based on ODD typologies, whether it be oppositionality and irritability or headstrong, hurtful, and irritable behavior, is unrealistic. Furthermore, in contrast to considering the mere presence of ODD symptoms, an approach which incorporated ODD symptom severity, duration, and impairment resulted in a viable class differentiation, that proved stable across age and gender, suggesting that these can be identified through childhood and adolescence, and in girls and boys.

There are several, partially overlapping, explanations why the present study failed to find ODD classes which were solely high in irritability (irritable ODD class) or solely high in oppositionality (oppositional ODD class). First, data-driven studies in clinic-referred boys^15^ and detained male adolescents,^16^ which found oppositional and irritable ODD classes, were relatively underpowered for the LCAs performed.^15^ Hence, it cannot be excluded that these classes emerged as a chance finding. Second too many patients may display irritability (e.g., those with major depressive disorder), oppositionality (e.g., those with ASD), or both (e.g., those with ODD), thereby restricting the likelihood to find Irritable ODD and oppositional ODD classes. Third, the strong correlation between irritability and oppositionality in our study (*r* = 0.62, see Supplement 1) might explain why only classes of increasing severity emerged.

Importantly, this overall increase in ODD symptom severity also indicates that other proposed subtyping approaches of ODD,^9,32^ including the *DSM*’s differentiation between angry/irritable mood, defiant/headstrong behavior and vindictiveness,^1^ as well as the ICD’s distinction between ODD with chronic irritability-anger and ODD without chronic irritability-anger,^33^ are unsuitable to classify individuals into mutually exclusive groups or classes. In addition, our results also deny the existence of a theoretically proposed ODD class comprised of youths with predominantly noncompliant symptoms and without anger and irritability.^19^ However, aside from classification, the ODD dimensions’ distinct correlates can still provide some clinical relevance. For example, irritability is mainly associated with affective problems, while oppositionality correlates with ADHD, CD, and delinquency.^7,8^ In sum, our results do raise the question to what extent distinct diagnostic groups in a psychiatric setting can be found that merely display one type of ODD behavior.

Rather, we found indications that besides serving as a differentiating characteristic, overall ODD symptom severity may serve as a guidance for ODD treatment. The high ODD class, overall, showed the highest levels of concurrent parent-, teacher- and/or self-reported hyperactivity, peer, and total mental health problems, and lower levels of prosocial behavior, followed by the moderate and low classes. With regard to DAWBA computer-generated classifications at referral, the high ODD class showed higher rates of ODD, CD, and ADHD than the 2 other classes and higher rates of GAD and ASD than the Low ODD class. Although fewer differences emerged between moderate and low ODD classes, youths in the moderate class were more troubled at referral in terms of dimensionally and categorically assessed mental health, and other problems. Altogether, the high ODD class constitutes the smallest class (26.4% of our sample) but appears to be the most troubled group at referral.

Importantly, the SDQ and computer-generated DAWBA classifications simply count the presence of problem behavior and cannot explain why symptoms occur (e.g., ODD symptoms as a manifestation of ODD or as a consequence of ASD). Clinicians are able to oversee different co-occurring symptoms and weigh their relative importance to one another. Therefore, it is crucial to test whether ODD classes differ in a meaningful manner when considering the clinician-rated and multidisciplinary team-based classifications at the end of the diagnostic process. Findings indicated higher rates of ODD and CD in the high ODD class compared to the other classes, which is not surprising since the ODD classes are based on ODD symptoms, while CD frequently co-occurs with ODD.^34,35^ The high ODD class also had the lowest levels of posttreatment functioning as measured by the GAF, followed by the moderate and low classes. Finally, the low ODD class had the highest rate of clinician-rated GAD classifications compared to the high and moderate ODD classes, and a higher rate of fear disorders compared to the high ODD class. Overall, this pattern of findings at the end of the diagnostic process contrasts with those at referral. This discrepancy may suggest that clinicians consider externalizing problems, like ODD or CD, to be the main problems of youths in the high ODD class. However, the discrepancy also indicates that, although externalizing problems are deemed the main problem in the high ODD class, affective problems are very prevalent. In sum, findings indicate that ODD classes based on low-cost questionnaires at referral are clearly predictive of clinically relevant outcomes as rated by clinicians months later. Interestingly, this study also shows that less severe ODD features at referral already bear prognostic usefulness. To illustrate, the moderate ODD class, consisting of youths with modest levels of ODD behaviors, showed considerable worse functioning compared to the low ODD class.

This study has several strengths: its large clinical sample, reliance on cross-sectional, and longitudinal data that were collected for applied clinical purposes, and its use of multiple informants. As always, there are several limitations. First, a part of the clinic-referred sample had no ODD-report available (790 excluded vs. 2185 included). Therefore, we cannot exclude a minor selection bias, for example, some parents did not meet the screening thresholds for the ODD questionnaire. This could make it relatively difficult to detect groups with one type of ODD behavior, like the irritable and oppositional classes. Nevertheless, considerable higher rates of ODD reports were available (73.4%) than regular referral rates because of behavioral problems (50%).^36,37^ Hence, we likely included the vast majority of youths with behavioral problems. Second, treatments were quite heterogeneous, and we were unable to collect reliable data on treatment engagement, intensity, and effectivity. Third, although our data-driven analytical approach greatly enables comparison with prior work, we did not explicitly test theory-driven approaches to account for heterogeneity among youths with ODD symptoms.^19^ Fourth, the data in this study were already available for a large sample. Clinicians who deal with children and their families at referral need to estimate to what ODD class a youth belongs, long before data are available for analyses within one’s own institution.

## Conclusion

This study indicates that youths who were high in irritability and oppositionality, were overall, most affected in terms of global functioning, concurrent and later mental health, and other problems. In contrast with prior work, our findings suggest that irritability and oppositionality in clinic-referred children and adolescents go hand in hand, making it improbable to assign individuals to classes which are only high in one of these behaviors.

## Supplemental Material

Supplemental Material, sj-rtf-1-cpa-10.1177_0706743720974840 - Classes of Oppositional Defiant Disorder Behavior in Clinic-referred Children and Adolescents: Concurrent Features and Outcomes: Classification Des Comportements Dans le Trouble Oppositionnel Avec Provocation Chez Des Enfants et des Adolescents Aiguillés à Une Clinique: Caractéristiques Co-occurrentes et Résultats Click here for additional data file. Supplemental Material, sj-rtf-1-cpa-10.1177_0706743720974840 for Classes of Oppositional Defiant Disorder Behavior in Clinic-referred Children and Adolescents: Concurrent Features and Outcomes: Classification Des Comportements Dans le Trouble Oppositionnel Avec Provocation Chez Des Enfants et des Adolescents Aiguillés à Une Clinique: Caractéristiques Co-occurrentes et Résultats by Peter J. Roetman, Berend M. Siebelink, Robert R. J. M. Vermeiren and Olivier F. Colins in The Canadian Journal of Psychiatry

Supplemental Material, sj-rtf-2-cpa-10.1177_0706743720974840 - Classes of Oppositional Defiant Disorder Behavior in Clinic-referred Children and Adolescents: Concurrent Features and Outcomes: Classification Des Comportements Dans le Trouble Oppositionnel Avec Provocation Chez Des Enfants et des Adolescents Aiguillés à Une Clinique: Caractéristiques Co-occurrentes et Résultats Click here for additional data file. Supplemental Material, sj-rtf-2-cpa-10.1177_0706743720974840 for Classes of Oppositional Defiant Disorder Behavior in Clinic-referred Children and Adolescents: Concurrent Features and Outcomes: Classification Des Comportements Dans le Trouble Oppositionnel Avec Provocation Chez Des Enfants et des Adolescents Aiguillés à Une Clinique: Caractéristiques Co-occurrentes et Résultats by Peter J. Roetman, Berend M. Siebelink, Robert R. J. M. Vermeiren and Olivier F. Colins in The Canadian Journal of Psychiatry

## Note

i. To facilitate comparison with prior work, especially with community samples, we also ran a latent class analysis (LCA) using a “symptom approach”, meaning that a very minimal threshold was used for an oppositional defiant disorder (ODD) symptom (i.e., “A little more than others”) to be present, without additional requirements for persistence and impairment. In short, this LCA solution, although stable across gender, did not result in very distinct ODD symptom profiles and was unstable across age (see Supplement 2; Table S5). Descriptive information and results from group comparisons of this LCA solution are available upon request.
