# Supplementary material for: Classes of Oppositional Defiant Disorder Behavior in Clinic-referred Children and Adolescents: Concurrent Features and Outcomes: Classification Des Comportements Dans le Trouble Oppositionnel Avec Provocation Chez Des Enfants et des Adolescents Aiguillés à Une Clinique: Caractéristiques Co-occurrentes et Résultats
Source: Can J Psychiatry. 2020 Nov 26;66(7):657–66. doi: 10.1177/0706743720974840 (PMC8240001; doi:10.1177/0706743720974840)
Supplement: Supplemental Material, sj-rtf-1-cpa-10.1177_0706743720974840 - Classes of Oppositional Defiant Disorder Behavior in Clinic-referred Children and Adolescents: Concurrent Features and Outcomes: Classification Des Comportements Dans le Trouble Oppositionnel Avec Provocation Chez Des Enfants et des Adol [file sj-rtf-1-cpa-10.1177_0706743720974840.rtf]

Table S1 
Oppositional Defiant Behavior Items of the Development and Well-Being Assessment (DAWBA)  	
Dimensions	Parent-version 	Teacher-version  	
Irritable	Had temper outbursts?	Temper tantrums or hot tempers	
	Been touchy or easily annoyed?	Easily annoyed by others	
	Been angry and resentful?	Angry and resentful	
Oppositional 	Seemed to do things to annoy other people on purpose?	Deliberately does things to annoy others	
	Blamed others for his/her own mistakes or bad behaviour?	Blames others for his/her own mistakes	
	Argued with grown-ups?	Argues a lot with adults	
	Taken no notice of rules, or refused to do as s/he is told?	Disobedient at school	
	Been spiteful?	Spiteful	
	Tried to get back at someone 	Tried to get back at someone	


Table S2
Steps in the Symptom-based ODD behavior Latent Class Analysis in Children Aged 5 through 11 (n = 1499) 	
Analysis	LL	BIC	BIC (sample adjusted)	AIC	Entropy	Bootstrap p-value	
1-class	-8973.139	18026.717	17991.773	17968.279			
2-class 	-6446.922	13040.095	12976.560	12933.844	0.847		
2-class (corrected for bivariate residuals) 	        Model did not converge					
2-class (corrected for bivariate residuals) no sex							
3-class	-6190.631	12607.951	12509.473	12443.262	0.822		
3-class (corrected for bivariate residuals)	-6101.663	12481.203	12360.488	12279.326	0.786		
3-class (corrected for bivariate residuals) no sex	-6105.268	12473.788	12359.426	12282.536	0.789	<.001	
4-class	-6148.064	12603.255	12469.833	12380.128	0.788		
4-class (corrected for bivariate residuals)	-6096.880	12537.450	12388.144	12287.760	0.760		
4-class (corrected for bivariate residuals) no sex	-6101.081	12523.915	12384.140	12290.163	0.752	<.001	
5-class	-6112.966	12613.498	12445.132	12331.933	0.752		
Note. AIC = Akaike Information Criterion; BIC = Bayesian Information Criterion; LL = Log-likelihood	


Table S3
Steps in the Symptom-based ODD behavior Latent Class Analysis in Children Aged 12  through 18 (n = 686) 	
Analysis	LL	BIC	BIC (sample adjusted)	AIC	Entropy	Bootstrap p-value	
1-class	-4200.938	8473.717	8438.790	8423.877			
2-class 	-2914.504	5959.626	5896.123	5869.008	0.865		
2-class (corrected for bivariate residuals) 	-2736.718	5675.893	5577.464	5535.436	0.868		
2-class (corrected for bivariate residuals) no sex	-2736.730	5669.387	5574.133	5533.461	0.866	<.001	
3-class	-2788.057	5778.571	5680.141	5638.114	0.838		
3-class (corrected for bivariate residuals)	-2720.288	5688.749	5568.093	5516.576	0.798		
3-class (corrected for bivariate residuals) no sex	-2720.447	5676.005	5561.700	5512.893	0.794	<.001	
4-class	-2756.396	5787.089	5653.733	5596.792	0.797		
4-class (corrected for bivariate residuals)							
4-class (corrected for bivariate residuals) no sex							
5-class	-2730.075	5806.287	5638.004	5566.150	0.821		
Note. AIC = Akaike Information Criterion; BIC = Bayesian Information Criterion; LL = Log-likelihood	


Table S4
Steps in the DSM-based ODD Latent Class Analysis 	
Analysis	LL	BIC	BIC (sample adjusted)	AIC	Entropy	Bootstrap p-value	
1-class	-19997.511	40094.984	40053.681	40021.022			
2-class 	-8823.071	17807.618	17740.898	17688.142	0.917		
2-class (corrected for bivariate residuals) 	        Model did not converge 					
2-class (corrected for bivariate residuals) age-only 						
2-class (corrected for bivariate residuals) sex-only						
2-class (corrected for bivariate residuals) no covariates						
3-class	-8159.108	16571.965	16467.119	16384.216	0.899		
3-class (corrected for bivariate residuals)	-7988.821	16339.042	16189.717	16071.642	0.832		
3-class (corrected for bivariate residuals) age-only  	-7995.788	16337.598	16194.627	16081.577	0.821		
3-class (corrected for bivariate residuals) sex-only	-7989.519	16325.059	16182.088	16069.037	0.820		
3-class (corrected for bivariate residuals) no covariates	-7996.763	16324.168	16187.551	16079.525	0.821	<.001	
4-class	-8067.291	16480.604	16337.633	16224.582	0.851		
4-class (corrected for bivariate residuals)	-7998.807	16389.771	16227.737	16099.614	0.835		
4-class (corrected for bivariate residuals) age-only  	-8005.390	16379.870	16227.368	16106.781	0.834		
4-class (corrected for bivariate residuals) sex-only	-7999.153	16367.396	16214.893	16094.306	0.833		
4-class (corrected for bivariate residuals) no covariates	-8006.650	16359.322	16216.351	16103.300	0.832	<.001	
5-class	-8015.251	16468.797	16287.700	16144.503	0.829		
5-class (corrected for bivariate residuals)	-7978.415	16433.571	16236.589	16080.830	0.816		
5-class (corrected for bivariate residuals) age-only	-7985.638	16417.259	16232.985	16087.275	0.805		
5-class (corrected for bivariate residuals) sex-only	-7982.488	16410.959	16226.685	16080.976	0.815		
5-class (corrected for bivariate residuals) no covariates	-7991.713	16398.652	16227.086	16091.426	0.808	<.001	
6-class	-7980.686	16491.939	16272.716	16099.372	0.827		
Note. AIC = Akaike Information Criterion; BIC = Bayesian Information Criterion; LL = Log-likelihood.	


Table S5
Steps in the Symptom-based ODD Behavior Latent Class Analysis 	
Analysis	LL	BIC	BIC (sample adjusted)	AIC	Entropy	Bootstrap p-value	
1-class	-18918.397	37936.757	37895.454	37862.795			
2-class 	-9412.654	18986.786	18920.066	18867.309	0.850		
2-class (corrected for bivariate residuals) 							
2-class (corrected for bivariate residuals) age-only 							
2-class (corrected for bivariate residuals) sex-only							
2-class (corrected for bivariate residuals) no covariates							
3-class	-9044.693	18343.135	18238.289	18155.386	0.816		
3-class (corrected for bivariate residuals)							
3-class (corrected for bivariate residuals) age-only  							
3-class (corrected for bivariate residuals) sex-only							
3-class (corrected for bivariate residuals) no covariates							
4-class	-8965.953	18277.927	18134.956	18021.906	0.861		
4-class (corrected for bivariate residuals)							
4-class (corrected for bivariate residuals) age-only  							
4-class (corrected for bivariate residuals) sex-only							
4-class (corrected for bivariate residuals) no covariates							
5-class	-8909.591	18257.476	18076.379	17933.181	0.792		
5-class (corrected for bivariate residuals)	-8832.732	18172.963	17963.272	17797.464	0.745		
5-class (corrected for bivariate residuals) age-only	-8837.797	18152.336	17955.353	17799.595	0.743	0.2174	
5-class (corrected for bivariate residuals) sex-only	-8863.570	18203.881	18006.898	17851.140	0.750		
5-class (corrected for bivariate residuals) no covariates	-8878.032	18202.048	18017.774	17872.065	0.708		
6-class	-8879.034	18288.634	18069.411	17896.067	0.775		
Note. AIC = Akaike Information Criterion; BIC = Bayesian Information Criterion; LL = Log-likelihood.	


Table S6
Means and Standard Deviations of the DSM-based ODD Classes on Highest Prevailing Parent- and Teacher-reported SDQ Scores	
	ODD classes	
	High ODD	Moderate ODD	Low ODD	
	(n = 565)	(n = 693)	(n = 906)	
SDQ Total Problems (SD)	23.29 (5.28)	20.51 (4.83)	18.28 (4.71)	
SDQ Emotional Problems (SD)	6.05 (2.57)	5.69 (2.55)	5.75 (2.50)	
SDQ Conduct Problems (SD)	5.75 (1.90)	4.21 (1.70)	3.28 (1.66)	
SDQ Hyperactivity (SD)	7.77 (2.14)	7.39 (2.25)	6.51 (2.53)	
SDQ Peer Problems (SD)	4.55 (2.20)	4.05 (2.28) 	3.55 (2.16)	
SDQ Prosocial (SD)	6.27 (1.99)	7.00 (1.97)	7.57 (1.83)	
Note. N = 2164. SDQ = strengths and difficulties questionnaire.	


Table S7
Prevalence of DAWBA Classifications of the DSM-based Oppositional Defiant Disorder Classes  	
	ODD classes 	
	High ODD	Moderate ODD	Low ODD	
	(n = 565)	(n = 693)	(n = 906)	
ODD [n(% of class)]	493 (87.3%)	388 (56.0%)	78 (8.6%)	
CD [n(% of class)]	154 (27.3%)	43 (6.2%)	22 (2.4%)	
ADHD [n(% of class)]	318 (56.3%)	314 (45.3%)	216 (23.8%)	
Depressive disorders [n(% of class)]	102 (18.1%)	93 (13.4%)	138 (15.2%)	
Generalized anxiety [n(% of class)]	114 (20.2%)	107 (15.4%)	134 (14.8%)	
Fear disorders [n(% of class)]	131 (23.2%)	136 (19.6%)	184 (20.3%)	
Autism spectrum disorders [n(% of class)]	39 (6.9%)	36 (5.2%)	24 (2.6%)	
Note. N = 2164. ADHD = attention deficit hyperactivity disorder; CD = conduct disorder; ODD = oppositional defiant disorder.	


Table S8
Prevalence of Clinical Classifications of the DSM-based Oppositional Defiant Disorder Classes 	
	ODD class 	
	High ODD	Moderate ODD	Low ODD	
	(n = 540)	(n = 653)	(n = 848)	
ODD [n(% of class)]	78 (14.4%)	57 (8.7%)	42 (5.0%)	
CD [n(% of class)]	41 (7.6%)	15 (2.3%)	13 (1.5%)	
ADHD [n(% of class)]	206 (38.1%)	249 (38.1%)	300 (35.4%)	
Depressive disorders [n(% of class)]	34 (6.3%)	38 (5.8%)	65 (7.7%)	
Generalized anxiety [n(% of class)]	13 (2.4%)	23 (3.5%)	56 (6.6%)	
Fear disorders [n(% of class)]	7 (1.3%)	15 (2.3%)	39 (4.6%)	
Autism spectrum disorder [n(% of class)]	131 (24.3%)	169 (25.9%)	186 (21.9%)	
Note. N = 2041. ADHD = attention deficit hyperactivity disorder; CD = conduct disorder; ODD = oppositional defiant disorder. 	
