# Supplementary material for: Classes of Oppositional Defiant Disorder Behavior in Clinic-referred Children and Adolescents: Concurrent Features and Outcomes: Classification Des Comportements Dans le Trouble Oppositionnel Avec Provocation Chez Des Enfants et des Adolescents Aiguillés à Une Clinique: Caractéristiques Co-occurrentes et Résultats
Source: Can J Psychiatry. 2020 Nov 26;66(7):657–66. doi: 10.1177/0706743720974840 (PMC8240001; doi:10.1177/0706743720974840)
Supplement: Supplemental Material, sj-rtf-2-cpa-10.1177_0706743720974840 - Classes of Oppositional Defiant Disorder Behavior in Clinic-referred Children and Adolescents: Concurrent Features and Outcomes: Classification Des Comportements Dans le Trouble Oppositionnel Avec Provocation Chez Des Enfants et des Adol [file sj-rtf-2-cpa-10.1177_0706743720974840.rtf]

Supplement 1
Detailed Measures
Clustering Variables
ODD symptoms were measured by the Dutch parent and teacher versions of the DAWBA, a widely-used computerized diagnostic interview which generates DSM-IV classifications. The parent version of the DAWBA has a gate-item which inquires if the child had exhibited any ODD-related symptoms in the last six months (i.e., “Not doing what they are told, being irritable or annoying, having temper outbursts, and so on”). The response on this gate item ranges from 0 (on average less difficult or problematic than other children), to 1 (about average) to 2 (on average more difficult or more problematic). If the parent endorses this gate-item with a 2, the ODD part of the DAWBA is activated, which inquires after the occurrence of the eight DSM-IV ODD symptoms in the last six months. The ODD part of the DAWBA is also activated when the parent indicates a score of 3 or higher on the SDQ conduct problems scale, which consists of five questions, and which is an integral part of the DAWBA. The teacher version of the DAWBA always directly asks teachers about all eight DSM-IV ODD symptoms. Of note, the Dutch version of the DAWBA separates the original DSM criterion of “vindictive and spiteful” into two different questions (see Table S1), resulting in a total of nine ODD symptoms. Assessment of impairment and persistence is considered a crucial diagnostic criterion for identifying individuals whose psychiatric disorders are of clinical significance. Therefore, the DAWBA also asks parents and teachers whether ODD symptoms have resulted in impairment in various developmental contexts (e.g., “Has his/her awkward behavior interfered with making and keeping friends”) and whether these symptoms have been present for more than 6 months. The impairment questions are rated from 0 (“Not at all”) to 3 (“A great deal”) and the persistence question is rated by 0 (“No”) or 1 (“Yes”).  
Whereas prior research on ODD classes merely considered if ODD symptoms were present or absent (from here onwards referred to as the symptom approach), the present study also considered symptom persistence (6 months ≤) and impairment (from here onwards referred to as the DSM approach). Specifically, according to this first approach an ODD symptom was coded as 1 (“behavior present”) when endorsed as 1 (“A little more than others”) or higher (2: “A lot more others”), while a score of 0 (“Not more often than others”) was dichotomized as 0 (“behavior absent”) (0 = 0; 1, 2 = 1). For the DSM approach, more stringent criterion for ODD presence were used; a symptom was coded as 1 (“behavior present”) when endorsed as 2 (“A lot more than others”), while lower scores were coded as 0 (“behavior absent”) (0, 1 = 0; 2 = 1). In addition, the reported ODD symptom was required to be present for six months or longer, and to cause impairment according to parent- and/or teacher-ratings. In both the symptom and DSM-approach parent- and teacher-ratings were combined by using highest prevailing scores (i.e., if at least one informant indicated an ODD behavior to be present, the behavior was indicated as present). Finally, the nine DAWBA ODD symptoms will be used as clustering variables in the person-oriented analyses (i.e., latent class analysis) to assign youths to mutually exclusive classes. 
External Variables for Cluster Comparisons: Concurrent Features at Referral
Dimensionally assessed mental health and other problems. The SDQ is a brief screening questionnaire that was completed as part of the DAWBA. The SDQ consists of 25 items which are scored on a 3-point Likert scale 0 (“not true”), 1 (“somewhat true”) and 2 (“certainly true”), and is subdivided in 5 subscales: Conduct Problems, Emotional Problems, Hyperactivity, Peer Problems, and Prosocial Behavior. The Total Problems scale consists of all SDQ items, minus the Prosocial Behavior scale. Because items of the Conduct Problems scale were used as gate items for the ODD symptoms, this scale was not used in class comparisons. Highest prevailing scores of parent-, teacher-, and, if applicable -youth self-report were used in the subsequent analyses. 
 Categorically assessed mental health problems. The DAWBA reports of parents, teachers, and youths who were at least 11 years of age, were used to generate computer-generated DSM classifications at referral. These classifications are based on predictions on the probability of the presence of various mental disorders. For each disorder, five categories are given, ranging from 0 (0.1 % of children in this category have the disorder in question) to 5 (70% of children in this category have the disorder in question). The categories were dichotomized into a “disorder absent” category ranging from values 0 to 3 (15% of children in this category have the disorder in question) and a “disorder present” category spanning values 4 (50% of children in this category have the disorder in question) and 5 [21]. To ease the interpretation of the results, and in line with previous recommendations [22], several DAWBA computer-generated DSM classifications were combined into disorder categories, from here onwards referred to as DAWBA computer-generated DSM disorder categories. Specifically, the category “depressive disorders” refers to the presence of major depressive disorder, dysthymic disorder, and/or depressive disorder not otherwise specified, whereas the category “fear disorders” refers to the presence of separation anxiety disorder, panic disorder, agoraphobia specific, and/or social phobia.
External Variables for Cluster Comparisons: Longitudinal Features 
Categorically assessed mental health problems. Psychiatric disorders, as defined by the DSM-IV, were determined at the end of a diagnostic process consisting of multidisciplinary psychiatric and psychological (semi-structured) evaluation conform clinical diagnostic guidelines by psychiatrists and psychologists. These evaluations took place on average 3.81 months (SD = 3.34) after completion of the DAWBA at referral. Any clinical classification, not just primary classifications, were included in the analyses. This was done to optimally use the classifications provided by the multidisciplinary team and because the DAWBA also provides multiple classifications per individual. From here onward, we refer to these disorders and disorder categories as Multidisciplinary Team-based Classifications of DSM Disorders and Disorder Categories.
Global functioning. DSM-based Global Assessment Functioning (GAF) scores give an indication of social, occupational, and psychological functioning of an individual, with a score of “100” indicating extremely high functioning, while “1” indicates severe impairment (e.g., persistent danger of severely hurting self or others, suicidal acts). General functioning of the youth at the beginning and end of treatment was measured through clinician-rated GAF scores. 


Supplement 2
Latent Class Analysis Model Selection 
The following steps were taken to select the best fitting latent class solution. First, models were selected on the basis of the Bayesian Information Criterion (BIC), which is considered to be the most reliable index of model fit in LCA after non-parametric bootstrapping.1 Although other indices of model fit were also studied, including: entropy, loglikelihood, Akaike information criterion (AIC). In the second step, to control for local independence, the Pearson chi-squared test of model fit was used to determine if main effects between items should be included in the model. When the Pearson chi-squared test of model fit indicated significance (p < .05), the item-pair with the highest bivariate residuals was included as a direct effect (e.g., the item-pair vindictive and spiteful), and the model rerun. This process was repeated until the Pearson chi-squared index indicated non-significance.2 The third step focussed on the influence of the covariates age and gender on the model, which was investigated by deleting the covariates in a stepwise manner. If exclusion of a covariate(s) resulted in a better model fit, the better fitting model was included in consequent analyses. The fourth step consisted of estimating model fit through non-parametric bootstrapping. The number of random starts perturbations varied per solution and was in each example increased until the best loglikelihood was replicated during the bootstrap runs. If a p-value was greater than .05 (indicating model fit) the model was chosen. When the p-value was lower than .05, the next most appropriate model was fitted, starting with step two. 
Symptom-based Latent Class Analysis: Stability of Age Covariate 
Because the symptom-based Latent Class Solution required age as a covariate, the robustness of age was investigated. This was done by running separate LCA's on two age groups: 11 years or younger (n = 1499), and 12 years or older (n = 686). Unfortunately, the five factor solution did not hold up with three classes found in the younger group and two-class solutions in the older group. Specifically, separate LCAs extracted three classes in children aged 11 or younger (n = 1499), and two classes in adolescents aged 12 or older (n = 686; see Tables S2-S3).
References
1. Nylund KL, Asparouhov T, Muthén BO. Deciding on the number of classes in latent class analysis and growth mixture modeling: A monte carlo simulation study. Structural equation modeling. 2007;14(4):535-569.
2. Asparouhov T, Muthén B. Residual associations in latent class and latent transition analysis. Structural Equation Modeling: A Multidisciplinary Journal. 2015;22(2):169-177.
